# Serum Uric Acid and Diabetic Peripheral Neuropathy in Type 2 Diabetes Mellitus: A Cross‐Sectional Study From Northeastern Tanzania

**DOI:** 10.1155/jdr/4174684

**Published:** 2026-08-06

**Authors:** Raya S. Hamad, William P. Howlett, Abid M. Sadiq, Isaack A. Lyaruu, Hudaa F. Akrabi, Elifuraha W. Mkwizu, Kajiru G. Kilonzo, Furaha S. Lyamuya, Gissela B. Nyakunga, Elichilia R. Shao, Daniel P. Mujuni, Damasi F. Bayo, Nattasha Mattaka, Phibi Ongujo, Ng’wamba Ntale, Leanji Leonard, Henry L. Mlay, Nyasatu G. Chamba

**Affiliations:** ^1^ Department of Internal Medicine, KCMC University, Moshi, Tanzania, kcmuco.ac.tz; ^2^ Department of Internal Medicine, Kilimanjaro Christian Medical Centre, Moshi, Tanzania, kcmc.ac.tz; ^3^ Management and Development for Health, Dar es Salaam, Tanzania

**Keywords:** diabetes mellitus Type 2, diabetic neuropathies, hyperuricemia, Tanzania, Toronto Clinical Scoring System

## Abstract

**Background:**

Diabetic peripheral neuropathy (DPN) is a common and disabling complication of Type 2 diabetes mellitus (T2DM). Emerging evidence suggests that elevated serum uric acid (SUA) may associate with microvascular complications; however, data from sub‐Saharan Africa remain limited. We examined the association between hyperuricemia and DPN among adults with T2DM in northern Tanzania.

**Methods:**

We conducted a hospital‐based cross‐sectional analytical study at a tertiary diabetes clinic from November 2024 to April 2025. Adults aged ≥ 18 years with established T2DM were consecutively recruited. DPN was assessed using the Toronto Clinical Scoring System (TCSS), with DPN defined as TCSS ≥ 6. Fasting SUA was classified as elevated at ≥ 340 *μ*mol/L in women and ≥ 420 *μ*mol/L in men. Multivariable logistic regression estimated adjusted odds ratios (aORs). Receiver operating characteristic (ROC) analysis assessed discriminatory performance.

**Results:**

Of 321 clinic attendees, 220 participants met eligibility criteria and were analyzed (mean age < 65 years: 69.1%; male: 53.2%). DPN prevalence was 51.8% (114/220). Elevated SUA was present in 52.7% and was strongly associated with DPN (aOR 5.89, 95% CI 3.01–11.51, *p* < 0.001). Diabetes duration ≥ 5 years (aOR 3.81, *p* = 0.016), overweight status (aOR 2.18, *p* = 0.034), elevated fasting glucose (aOR 2.20, *p* = 0.022), and low HDL (aOR 2.10, *p* = 0.040) were independently associated with DPN. Elevated total cholesterol was inversely associated (aOR 0.36, *p* = 0.003). SUA demonstrated moderate discrimination for DPN (AUC 0.773).

**Conclusions:**

Hyperuricemia was independently associated with prevalent DPN in this Tanzanian study. SUA may represent a clinically accessible marker for neuropathy risk stratification in resource‐limited settings.

## 1. Introduction

Diabetes mellitus is a major global public health challenge, with an estimated 537 million adults affected worldwide in 2021, a number projected to increase to 783 million by 2045 [1]. The burden is rising most rapidly in low‐ and middle‐income countries (LMICs), particularly in sub‐Saharan Africa (SSA), where urbanization, population aging, and lifestyle changes have contributed to increasing prevalence [2]. Tanzania reflects this trend, with a growing number of individuals living with Type 2 diabetes mellitus (T2DM) and consequently at increased risk of chronic microvascular complications [3].

Among these complications, diabetic peripheral neuropathy (DPN) represents one of the most common and disabling manifestations. DPN affects up to 50% of individuals with long‐standing diabetes and constitutes the leading cause of nontraumatic lower limb amputation worldwide [4, 5]. It contributes substantially to foot ulceration, falls, chronic pain, reduced mobility, impaired quality of life, and increased mortality [4–6]. The American Diabetes Association (ADA) and international consensus statements recognize DPN as a symmetrical, length‐dependent sensorimotor polyneuropathy attributable to chronic hyperglycemia and associated metabolic disturbances [4]. However, its pathogenesis extends beyond chronic hyperglycemia alone.

DPN arises from a complex interplay of metabolic, vascular, inflammatory, and oxidative mechanisms. Chronic hyperglycemia activates the polyol pathway, increases advanced glycation end products, enhances protein kinase C activity, and stimulates hexosamine pathway flux, collectively promoting oxidative stress and mitochondrial dysfunction [7]. Simultaneously, endothelial dysfunction impairs microvascular blood flow to peripheral nerves, leading to ischemia and axonal injury [7]. Dyslipidemia, hypertension, and insulin resistance further amplify these processes. Collectively, these mechanisms indicate that DPN results from complex metabolic and vascular injury rather than chronic hyperglycemia alone [6, 7].

In recent years, serum uric acid (SUA) has emerged as a potential contributor to cardiometabolic and microvascular disease. Uric acid represents the final product of purine metabolism in humans and is generated primarily through xanthine oxidase activity. Although physiological extracellular uric acid exhibits antioxidant properties, elevated intracellular uric acid may induce oxidative stress, endothelial dysfunction, inflammation, and nitric oxide depletion [8, 9]. Experimental studies demonstrate that uric acid stimulates NADPH oxidase activity, activates the NLRP3 inflammasome, and promotes vascular smooth muscle proliferation, thereby impairing microvascular integrity [8, 9]. These mechanisms plausibly intersect with pathways implicated in peripheral nerve ischemia and axonal degeneration.

Epidemiological studies have linked hyperuricemia with hypertension, chronic kidney disease (CKD), metabolic syndrome, and T2DM [10, 11]. A large prospective cohort from the Rotterdam Study demonstrated that elevated SUA predicted incident T2DM independent of traditional risk factors [10]. Similarly, systematic reviews confirm that hyperuricemia clusters with insulin resistance and systemic inflammation [11]. These observations suggest that SUA may contribute to the development of DPN beyond its association with traditional cardiometabolic risk factors.

Several studies from Asia and Europe have reported positive associations between SUA and DPN, whereas evidence from SSA remains limited. Studies from Ethiopia and Sudan report high prevalence rates of DPN among adults with T2DM, ranging from 40% to 60%, depending on diagnostic methods [12, 13]. In rural Uganda, Munyambalu et al. documented a DPN prevalence exceeding 50% using clinical screening tools [14]. However, few African studies have specifically examined SUA in relation to neuropathic complications. Research from Tanzania has primarily focused on hyperuricemia prevalence and its association with metabolic syndrome rather than microvascular outcomes [15]. Consequently, the relationship between SUA and DPN in East African populations remains insufficiently characterized.

This knowledge gap is clinically important. SSA populations often present with prolonged undiagnosed diabetes, limited access to specialist care, a high hypertension burden, and constrained diagnostic resources [2, 3, 14]. Electrophysiological testing is rarely available in routine diabetes care across many resource‐limited settings. Clinical tools such as the Toronto Clinical Scoring System (TCSS) provide pragmatic alternatives for DPN diagnosis in resource‐limited settings [16]. Understanding modifiable metabolic correlates of DPN within this context could inform risk stratification and preventive strategies.

Therefore, we investigated the association between sex‐specific SUA levels and DPN among adults with T2DM attending a tertiary diabetes clinic in northern Tanzania. We hypothesized that hyperuricemia would be independently associated with prevalent DPN after adjustment for established clinical risk factors. By addressing an important evidence gap in SSA, this study provides clinically relevant data on the potential role of SUA as an accessible biomarker for neuropathy risk stratification in resource‐limited settings.

## 2. Methods

### 2.1. Study Design and Setting

We conducted a hospital‐based cross‐sectional analytical study at the Diabetes Clinic of Kilimanjaro Christian Medical Centre (KCMC), a tertiary referral hospital in northern Tanzania. The study was carried out over a 6‐month period from November 1, 2024, to April 30, 2025. Participants were recruited consecutively during routine outpatient clinic visits to minimize selection bias.

### 2.2. Study Population

#### 2.2.1. Inclusion Criteria

We included adults aged ≥ 18 years with an established diagnosis of T2DM. T2DM status was confirmed through documented clinical diagnosis in medical records. No new diagnoses were made during the study period.

#### 2.2.2. Exclusion Criteria

We excluded individuals with age > 75 years, pregnant women, those with CKD with estimated glomerular filtration rate (eGFR) < 30 mL/min/1.73 m^2^, those with severe anemia, defined according to World Health Organization (WHO) criteria (hemoglobin < 8 g/dL) [17], those with confirmed malignancies, human immunodeficiency virus infection, and use of medications known to significantly affect SUA levels (e.g., urate‐lowering therapy, high‐dose diuretics, and cytotoxic agents). These exclusions were applied to reduce confounding factors that may independently influence neuropathy or SUA levels.

### 2.3. Sample Size Determination

We calculated the minimum required sample size using the formula for comparison of two proportions in cross‐sectional studies. The calculation was based on hyperuricemia prevalence among patients with DPN (56%) [18], hyperuricemia prevalence among patients without DPN (36%) [19], 80% power, and 95% confidence level.

The minimum required sample size was 104 participants per group, yielding a total of 208 participants. We enrolled 220 participants to account for potential incomplete data and ensure adequate statistical power.

### 2.4. Data Collection Procedures

After obtaining written informed consent, trained clinicians collected sociodemographic, clinical, and anthropometric data using a structured data collection form.

#### 2.4.1. Anthropometric Measurements

Body weight and height were measured using standardized equipment. Body mass index (BMI) was calculated as weight (kilogram)/height (square meter) and categorized according to WHO criteria [20].

#### 2.4.2. Blood Pressure (BP) Measurement

BP was measured after at least 5 min of rest using a calibrated sphygmomanometer. Elevated BP was defined as systolic BP ≥ 130 mmHg and/or diastolic BP ≥ 80 mmHg, consistent with recommendations for individuals with diabetes [21].

#### 2.4.3. Laboratory Measurements

Participants underwent fasting venous blood sampling after an overnight fast of at least 8 h. SUA was measured using the enzymatic uricase–peroxidase method on a Roche Cobas automated chemistry analyzer. Hyperuricemia was defined using sex‐specific cutoffs: ≥ 340 *μ*mol/L in women and ≥ 420 *μ*mol/L in men [22]. Fasting blood glucose (FBG) was categorized using ADA criteria [23] with normal (≤ 7.0 mmol/L) and elevated (> 7.0 mmol/L). Glycated hemoglobin (HbA1c) was categorized as controlled (≤ 7%) and poor control (> 7%) [23].

Serum creatinine was measured using an enzymatic method. eGFR was calculated using the CKD‐EPI equation [24]. Lipid parameters were categorized based on cutoffs as follows [25]: total cholesterol (> 5.2 mmol/L), low‐density lipoprotein cholesterol (LDL‐C) (> 2.6 mmol/L), high‐density lipoprotein cholesterol (HDL‐C) (< 1.03 mmol/L), and triglycerides (> 1.7 mmol/L). Hemoglobin levels were classified using WHO criteria [17].

#### 2.4.4. Definition and Assessment of DPN

DPN was assessed using the TCSS, a validated clinical instrument for diagnosing diabetic neuropathy [16]. The TCSS is a standardized 19‐point scale comprising neuropathic symptoms (maximum 6 points), sensory testing (maximum 5 points), and deep tendon reflexes (maximum 8 points). The total possible score ranges from 0 to 19. DPN was defined as a TCSS score ≥ 6, consistent with validated diagnostic thresholds [16]. The TCSS assessment was performed by trained clinicians before SUA results were disclosed to minimize measurement bias. Interobserver variability was assessed during pilot testing to ensure consistency.

### 2.5. Statistical Analysis

Data were analyzed using SPSS Version 26. Continuous variables were summarized as means ± standard deviation (SD) or medians with interquartile ranges (IQRs), depending on distribution. Categorical variables were summarized as frequencies and percentages. We performed univariate logistic regression to calculate crude odds ratios (cORs) and 95% confidence intervals (CIs). Variables with *p* < 0.20 in univariate analysis were entered into multivariable logistic regression to compute adjusted odds ratios (aORs). Statistical significance was defined as *p* < 0.05. We generated ROC curves to evaluate the discriminatory ability of continuous SUA and continuous TCSS for identifying DPN. The area under the curve (AUC) with 95% CI was calculated. The optimal cutoff value was determined using the Youden index (*J* = sensitivity + specificity − 1).

### 2.6. Ethical Considerations

The Kilimanjaro Christian Medical University College Research and Ethics Committee approved the study (Approval No. PG 059/2024). Administrative permission was obtained from hospital leadership prior to study initiation. Participants received detailed study information and provided written informed consent. Participation was voluntary, and refusal did not affect clinical care. Data were anonymized using unique identification numbers and stored securely to maintain confidentiality.

## 3. Results

During the study period, 321 patients attended the diabetes clinic. Of these, 309 consented and were screened for eligibility. Seventy‐one patients were excluded based on predefined criteria. A total of 220 participants met the eligibility criteria and were included in the final data collection and analysis.

Table 1 presents the sociodemographic characteristics of the study population. Most participants were younger than 65 years (69.1%), while 68 (30.9%) were aged ≥ 65 years. Males comprised 117 (53.2%) of the study and females 103 (46.8%). The majority resided in urban areas (65.0%), were married (69.1%), and were not earning income (55.5%). Slightly more than half had primary education or less (51.4%). Recent alcohol intake was reported by 25 participants (11.4%), and 32 (14.5%) had a history of smoking. Most participants consumed fewer than 2.5 servings of meat per day (84.5%), fewer than 2 servings of dairy per day (78.6%), and ≤ 0.8 servings of fish per day (85.5%).

**Table 1:** Sociodemographic characteristics of patients with Type 2 diabetes mellitus (*n* = 220).

Variables	*n*	%
Age (years); mean (±SD): 61 (10)		
≥ 65	68	30.9
< 65	152	69.1
Sex		
Male	117	53.2
Female	103	46.8
Address		
Urban	143	65
Rural	77	35
Marital status		
Married	152	69.1
Not married	68	30.9
Employment status		
Earning	98	44.5
Not earning	122	55.5
Education status		
Primary level and less	113	51.4
Secondary level and more	107	48.6
Recent alcohol intake		
Yes	25	11.4
No	195	88.6
Smoking history		
Yes	32	14.5
No	188	85.5
Meat servings per day		
≥ 2.5	34	15.5
< 2.5	186	84.5
Dairy servings per day		
≥ 2	47	21.4
< 2	173	78.6
Fish servings per day		
≤ 0.8	188	85.5
> 0.8	32	14.5

Table 2 summarizes the clinical and laboratory characteristics of the study population. Most participants had diabetes duration ≥ 5 years (88.2%) and were receiving oral hypoglycemic agents alone (56.4%). Hyperuricemia was present in 116 participants (52.7%), while elevated FBG and HbA1c were observed in 50.0% and 53.6% of participants, respectively. Based on the TCSS, 114 participants (51.8%) met the diagnostic criteria for DPN, including 53 (24.1%) with mild, 44 (20.0%) with moderate, and 17 (7.7%) with severe neuropathy. Overall, 169 (76.8%) participants were receiving antihypertensives, while 92 (41.8%) were receiving statins.

**Table 2:** Clinical and laboratory characteristics of patients with Type 2 diabetes mellitus (*n* = 220).

Variables	*n*	%
Duration of diabetes (years); mean (±SD): 13.5 (7.2)		
≥ 5	194	88.2
< 5	26	11.8
Diabetes medications		
Insulin	45	20.5
Insulin and OHA	51	23.2
OHA	124	56.4
BMI (kg/m^2^); mean (±SD): 28.9 (5.2)		
Obese (≥ 30)	26	11.8
Overweight (25.0–29.9)	82	37.3
Underweight (< 18.5)	15	6.8
Normal (18.5–24.9)	97	44.1
Systolic blood pressure (mmHg); mean (±SD): 144.6 (22.4)		
Elevated (≥ 140)	187	85
Normal (< 140)	33	15
Diastolic blood pressure (mmHg); mean (±SD): 91.8 (10.3)		
Elevated (≥ 90)	150	68.2
Normal (< 90)	70	31.8
Serum uric acid levels		
Elevated	116	52.7
Normal	104	47.3
Fasting blood glucose levels (mmol/L); mean (±SD): 7.1 (1.2)		
> 7.1	110	50
≤ 7.1	110	50
HbA1C levels (%); mean (±SD): 8.5 (2.5)		
> 7	118	53.6
≤ 7	102	46.4
eGFR levels (mL/min/1.73 m^2^); mean (±SD): 73 (12)		
< 60	39	17.7
≥ 60	181	82.3
Hemoglobin levels (g/dL); mean (±SD): 12.5 (3.5)		
≤ 10	80	36.4
> 10	140	63.6
Total cholesterol levels (mmol/L); mean (±SD): 5.0 (1.4)		
Elevated (> 5.2)	89	40.5
Normal (≤ 5.2)	131	59.5
LDL‐C levels (mmol/L); mean (±SD): 2.4 (1.1)		
Elevated (> 2.6)	34	15.5
Normal (≤ 2.6)	186	84.5
HDL‐C levels (mmol/L); mean (±SD): 1.4 (0.9)		
Reduced (< 1.03)	75	34.1
Normal (≥ 1.03)	145	65.9
Triglyceride levels (mmol/L); mean (±SD): 1.4 (0.5)		
Elevated (> 1.7)	64	29.1
Normal (≤ 1.7)	156	70.9
TCSS		
Severe (≥ 12)	17	7.7
Moderate (9–11)	44	20
Mild (6–8)	53	24.1
No (0–5)	106	48.2
Diabetic neuropathy		
Yes	114	51.8
No	106	48.2

Abbreviations: BMI, body mass index; eGFR, estimated glomerular filtration rate; HbA1C, glycated hemoglobin; HDL‐C, high‐density lipoprotein cholesterol; LDL‐C, low‐density lipoprotein cholesterol; OHA, oral hypoglycemic agents; TCSS, Toronto Clinical Scoring System.

**Figure 1:**
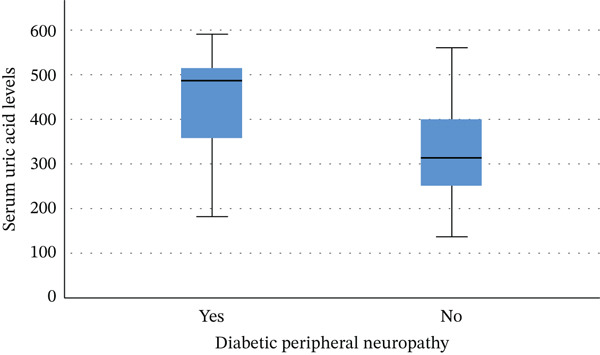
Boxplot illustrates serum uric acid levels against diabetic peripheral neuropathy.

Figure 1 illustrates the distribution of SUA levels according to DPN status. Participants with DPN demonstrated a markedly higher median SUA level (approximately 480–490 *μ*mol/L) compared with those without DPN (approximately 310–320 *μ*mol/L), with a clear upward shift in the IQR among individuals with neuropathy.

**Figure 2:**
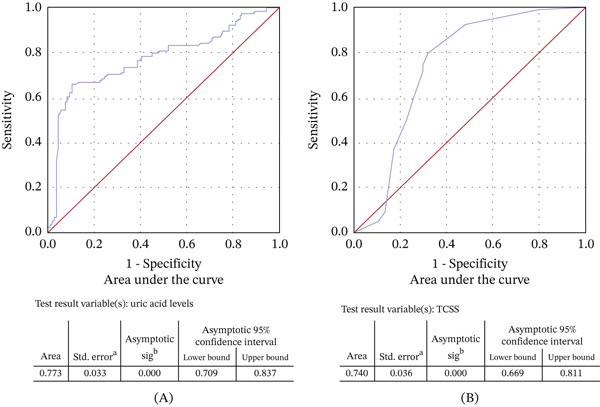
ROC analysis showing (A) serum uric acid levels against diabetic peripheral neuropathy and (B) TCSS against hyperuricemia.

Receiver operating characteristic analysis of continuous SUA (Figure 2A) yielded an AUC of 0.773 (95% CI 0.709–0.837, *p* < 0.001), indicating moderate discriminatory ability for identifying DPN. ROC analysis of continuous TCSS (Figure 2B) produced an AUC of 0.740 (95% CI 0.669–0.811, *p* < 0.001). Both measures demonstrated statistically significant discriminatory ability for identifying DPN, with SUA yielding a numerically higher AUC than the TCSS.

Table 3 compares participants with and without DPN. Among those with DPN, 36 (31.6%) were aged ≥ 65 years compared with 32 (30.2%) among those without DPN (cOR 1.07, 95% CI 0.60–1.89, *p* = 0.82). Male sex was not significantly associated with DPN (cOR 1.19, 95% CI 0.70–2.02, *p* = 0.52). Urban residence, marital status, employment status, education level, alcohol intake, smoking history, and dietary variables were not significantly associated with DPN.

**Table 3:** Regression analysis of patients with diabetic peripheral neuropathy among Type 2 diabetes mellitus.

Variables	DPN		cOR (95% CI)	*p* value	aOR (95% CI)	*p* value
	Yes	No				
Age (years)						
≥ 65	36 (31.6)	32 (30.2)	1.07 (0.60–1.89)	0.82		
< 65	78 (68.4)	74 (69.8)	1			
Sex						
Male	63 (55.3)	54 (50.9)	1.19 (0.70–2.02)	0.52		
Female	51 (44.7)	52 (49.1)	1			
Address						
Urban	78 (68.4)	65 (61.3)	1.37 (0.78–2.38)	0.27		
Rural	36 (31.6)	41 (38.7)	1			
Marital status						
Married	82 (71.9)	70 (66.0)	1.32 (0.74–2.34)	0.35		
Not married	32 (28.1)	36 (34.0)	1			
Employment status						
Earning	50 (43.9)	48 (45.3)	0.94 (0.55–1.61)	0.83		
Not earning	64 (56.1)	58 (54.7)	1			
Education status						
Primary level and less	59 (51.8)	54 (50.9)	1.03 (0.61–1.75)	0.9		
Secondary level and more	55 (48.2)	52 (49.1)	1			
Recent alcohol intake						
Yes	13 (11.4)	12 (11.3)	1.01 (0.44–2.32)	0.99		
No	101 (88.6)	94 (88.7)	1			
Smoking history						
Yes	15 (13.2)	17 (16.0)	0.79 (0.37–1.68)	0.55		
No	99 (86.8)	89 (84.0)	1			
Meat servings per day						
≥ 2.5	15 (13.2)	19 (17.9)	0.69 (0.33–1.45)	0.33		
< 2.5	99 (86.8)	87 (82.1)	1			
Dairy servings per day						
≥ 2	28 (24.6)	19 (17.9)	1.49 (0.78–2.87)	0.23		
< 2	86 (75.4)	87 (82.1)	1			
Fish servings per day						
≤ 0.8	98 (86.0)	90 (84.9)	1.09 (0.52–2.31)	0.82		
> 0.8	16 (14.0)	16 (15.1)	1			
Duration of diabetes (years)						
≥ 5	106 (93.0)	88 (83.0)	2.71 (1.13–6.53)	0.022	3.81 (1.29–11.28)	0.016
< 5	8 (7.0)	18 (17.0)	1			
Diabetes medications						
Insulin	25 (21.9)	20 (18.9)	1.00 (0.50–1.98)	0.99	0.90 (0.39–2.07)	0.8
Insulin and OHA	20 (17.5)	31 (29.2)	0.51 (0.27–1.00)	0.05	0.42 (0.18–0.98)	0.044
OHA	69 (60.5)	55 (51.9)	1			
BMI						
Obese	16 (14.0)	10 (9.4)	2.38 (0.98–5.78)	0.056	2.44 (0.81–7.35)	0.114
Overweight	49 (43.0)	33 (31.1)	2.21 (1.21–4.02)	0.01	2.18 (1.06–4.50)	0.034
Underweight	10 (8.8)	5 (4.7)	2.97 (0.94–9.37)	0.063	2.48 (0.62–9.80)	0.196
Normal	39 (34.2)	58 (54.7)	1			
Systolic blood pressure						
Elevated	98 (86.0)	89 (84.0)	1.17 (0.56–2.45)	0.68		
Normal	16 (14.0)	17 (16.0)	1			
Diastolic blood pressure						
Elevated	81 (71.1)	69 (65.1)	1.32 (0.75–2.32)	0.34		
Normal	33 (28.9)	37 (34.9)	1			
Serum uric acid levels						
Elevated	83 (72.8)	33 (31.1)	5.92 (3.31–10.60)	< 0.001	5.89 (3.01–11.51)	< 0.001
Normal	31 (27.2)	73 (68.9)	1			
Fasting blood glucose levels (mmol/L)						
> 7.1	67 (58.8)	43 (40.6)	2.09 (1.22–3.58)	0.007	2.20 (1.12–4.31)	0.022
≤ 7.1	47 (41.2)	63 (59.4)	1			
HbA1C levels (%)						
> 7	59 (51.8)	59 (55.7)	0.86 (0.50–1.45)	0.56		
≤ 7	55 (48.2)	47 (44.3)	1			
eGFR levels (mL/min/1.73 m^2^)						
< 60	22 (19.3)	17 (16.0)	1.25 (0.62–2.51)	0.53		
≥ 60	92 (80.7)	89 (84.0)	1			
Hemoglobin levels (g/dL)						
≤ 10	42 (36.8)	38 (35.8)	1.04 (0.60–1.81)	0.88		
> 10	72 (63.2)	68 (64.2)	1			
Total cholesterol levels (mmol/L)						
> 5.2	35 (30.7)	54 (50.9)	0.43 (0.25–0.74)	0.002	0.36 (0.18–0.71)	0.003
≤ 5.2	79 (69.3)	52 (49.1)	1			
LDL levels (mmol/L)						
> 2.6	14 (12.3)	20 (18.9)	0.60 (0.29–1.26)	0.177	0.56 (0.22–1.44)	0.228
≤ 2.6	100 (87.7)	86 (81.1)	1			
HDL levels (mmol/L)						
< 1.03	52 (45.6)	23 (21.7)	3.03 (1.68–5.47)	< 0.001	2.10 (1.04–4.25)	0.04
≥ 1.03	62 (54.4)	83 (78.3)	1			
Triglyceride levels (mmol/L)						
> 1.7	35 (30.7)	29 (27.4)	1.18 (0.66–2.11)	0.59		
≤ 1.7	79 (69.3)	77 (72.6)	1			

*Note:* Multivariable logistic regression included variables with *p* < 0.20 in the univariate analysis: duration of diabetes, diabetes medications, body mass index, serum uric acid, fasting blood glucose, LDL‐C, HDL‐C, and total cholesterol.

Abbreviations: BMI, body mass index; eGFR, estimated glomerular filtration rate; HbA1c, glycated hemoglobin; HDL‐C, high‐density lipoprotein cholesterol; LDL‐C, low‐density lipoprotein cholesterol; OHA, oral hypoglycemic agents.

Diabetes duration ≥ 5 years was significantly associated with DPN, occurring in 106 of 114 participants with DPN (93.0%) compared with 88 of 106 without DPN (83.0%) (cOR 2.71, 95% CI 1.13–6.53, *p* = 0.022). After multivariable adjustment, diabetes duration ≥ 5 years remained independently associated with DPN (aOR 3.81, 95% CI 1.29–11.28, *p* = 0.016). Compared with oral hypoglycemic agents alone, combination therapy with insulin and oral agents was independently associated with lower odds of DPN (aOR 0.42, 95% CI 0.18–0.98, *p* = 0.044). Overweight status was independently associated with DPN (aOR 2.18, 95% CI 1.06–4.50, *p* = 0.034), whereas obesity did not retain statistical significance after adjustment.

Elevated SUA was strongly associated with DPN. Among participants with DPN, 83 (72.8%) had elevated SUA compared with 33 (31.1%) among those without DPN (cOR 5.92, 95% CI 3.31–10.60, *p* < 0.001). This association remained robust after adjustment for confounders (aOR 5.89, 95% CI 3.01–11.51, *p* < 0.001). Elevated FBG was also independently associated with DPN (aOR 2.20, 95% CI 1.12–4.31, *p* = 0.022), whereas HbA1c was not significantly associated (*p* = 0.56). Reduced eGFR and hemoglobin levels were not significantly associated with DPN. Elevated total cholesterol was less frequent among participants with DPN (35/114, 30.7%) than among those without DPN (54/106, 50.9%) and was independently associated with lower odds of DPN (aOR 0.36, 95% CI 0.18–0.71, *p* = 0.003). Low HDL cholesterol (< 1.03 mmol/L) was independently associated with DPN (aOR 2.10, 95% CI 1.04–4.25, *p* = 0.040). LDL cholesterol and triglycerides were not significantly associated with DPN.

## 4. Discussion

In this study, we found that sex‐specific hyperuricemia (≥ 340 *μ*mol/L in women and ≥ 420 *μ*mol/L in men) was common (52.7%) and strongly associated with prevalent DPN. After adjustment for demographics, diabetes duration, glycemic measures, renal function, lipids, BMI, and other covariates, elevated SUA conferred nearly a six‐fold higher odds of DPN (aOR 5.89, *p* < 0.001). In addition, diabetes duration ≥ 5 years, overweight status, elevated fasting glucose, and low HDL are independently associated with DPN in our study. Continuous SUA demonstrated moderate discrimination for prevalent DPN (AUC 0.773). These findings contribute new, region‐specific evidence to a growing but heterogeneous literature on uric acid and diabetic neuropathy and carry both mechanistic and clinical implications.

Our DPN prevalence (51.8%) is consistent with pooled estimates from Africa, which place clinic‐based DPN prevalence in similar high‐burden ranges (approximately 40%–50%), and exceeds typical rates reported from many high‐income country studies where prevalence estimates are often lower when using population‐based samples and electrophysiology [26]. The high proportion of participants with long diabetes duration (88% ≥ 5 years), moderate rates of poor glycemic control (FBG > 7.0 mmol/L in 50% and HbA1c > 7*%* in 53.6%), and a high burden of hypertension in our sample likely contributed to the elevated DPN prevalence and are typical of tertiary clinic populations in LMICs [27].

Our principal result, a strong, independent association between elevated SUA and DPN, parallels multiple observational reports but is larger in magnitude than most published estimates. Several cross‐sectional studies in Asia and Europe have reported positive associations between SUA and peripheral neuropathy among people with T2DM, with adjusted effect sizes commonly in the 1.5–2.5 range [28–32]. Mendelian randomization and genetic correlation studies have recently suggested a possible causal link between genetically predicted urate and diabetic neuropathy, although causal inference remains under active investigation [33]. Our aOR (≈5.9) substantially exceeds prior estimates, which may reflect one or more of the following: (a) a higher prevalence and higher absolute SUA levels in our population (median SUA difference ≈170 *μ*mol/L between groups), (b) the concentration of advanced disease and comorbidity in a tertiary clinic with long diabetes duration, (c) the use of sex‐specific cutoffs that better capture biologic hyperuricemia in women and men, and (d) residual confounding or effect modification by unmeasured factors (dietary purine load, medication use, and unmeasured inflammatory markers). While prior studies have often adjusted for kidney function, the relatively low prevalence of eGFR < 60 (17.7%) in our sample and the persistence of the SUA effect after adjustment suggest that the SUA–DPN association is not fully explained by overt CKD in this study.

Although our findings support an association between elevated SUA and DPN, emerging evidence suggests that this relationship may be more complex. Uric acid possesses antioxidant properties under physiological conditions, and excessively low SUA concentrations may impair antioxidant defenses, potentially increasing susceptibility to oxidative nerve injury. Consequently, some studies have proposed a possible U‐shaped relationship between SUA and DPN, in which both low and high concentrations may be associated with adverse outcomes [32, 34]. Because our study focused on hyperuricemia using established sex‐specific thresholds, we were unable to evaluate the potential effects of low SUA levels, which warrant investigation in future prospective studies.

Our study also detected independent associations of DPN with diabetes duration ≥ 5 years, overweight status, elevated fasting glucose, and low HDL. The strong effect of duration aligns with long‐standing evidence that cumulative metabolic exposure raises neuropathy risk [4, 28]. That fasting glucose (but not HbA1c) associated with DPN in multivariable models may reflect the cross‐sectional snapshot of current metabolic burden, intraindividual variability in glycemic exposure over time, or limited ability of a single HbA1c to capture lifetime glycemic exposure in this population; similar discordances have been observed in other clinic‐based studies. Overweight (BMI 25–29.9 kg/m^2^) is associated with higher DPN odds, consistent with data linking adiposity, insulin resistance, and dyslipidemia to microvascular nerve injury [7, 30]. Low HDL retained an independent association, supporting the notion that atherogenic dyslipidemia and lipid‐driven endothelial dysfunction contribute to neuropathic pathways [30, 35].

Hyperuricemia frequently clusters with central obesity, insulin resistance, hypertension, and dyslipidemia and may therefore represent an additional marker of the adverse metabolic milieu associated with DPN, rather than an independent causal factor [11]. Consistent with this concept, overweight status and low HDL cholesterol were independently associated with DPN in our study, supporting the contribution of metabolic dysfunction to peripheral nerve injury [35]. In contrast, triglyceride levels were not independently associated with DPN despite their recognized role as a component of metabolic syndrome [35]. This discrepancy may reflect differences in study populations, sample size, use of lipid‐lowering therapy, or residual confounding, and similar inconsistencies have been reported in previous studies evaluating metabolic risk factors for DPN [30, 35].

The inverse association between elevated total cholesterol (>5.2 mmol/L) and DPN in our adjusted model is unexpected and merits careful interpretation. In our unadjusted descriptive data, elevated total cholesterol was more common among participants without DPN (50.9% vs. 30.7%), and this relationship persisted after adjustment (aOR 0.36). Several possible explanations exist. First, reverse confounding by statin therapy could contribute; patients with higher cardiovascular risk or dyslipidemia may receive more intensive lipid‐lowering therapy or closer clinical follow‐up, which could correlate with better neuropathy prevention or detection patterns; conversely, patients with advanced neuropathy may have lower appetite, malnutrition, or lipid levels reflecting illness severity. Second, aggressive lipid lowering (including lower total cholesterol) in some patients could reflect prior cardiovascular disease or frailty associated with neuropathy. Third, measurement or selection biases in a tertiary clinic population can produce paradoxical associations. Finally, residual confounding remains possible (e.g., dietary factors, inflammatory status, or medication use not fully captured) [31]. Because most prior studies either show neutral or positive associations between atherogenic lipids and neuropathy, we urge caution and recommend further prospective examination with medication data and temporally resolved lipid measures.

Clinically, elevated SUA may serve as an accessible biomarker to help stratify neuropathy risk in settings where electrophysiologic testing is not available. Because uric acid is modifiable through lifestyle interventions and urate‐lowering medications, our findings motivate prospective studies and randomized trials to determine whether lowering SUA reduces incident neuropathy or slows progression among people with T2DM, an approach that would require careful safety evaluation, especially in patients with CKD. Mendelian randomization and genetic correlation signals underscore the plausibility of a causal link but are not definitive; thus, interventional evidence is essential before recommending urate lowering for neuropathy prevention [33, 36]. At a health‐systems level, our results support integrated cardiometabolic care in LMIC clinics that addresses glycemic control, lipid disorders, adiposity, and potentially uric acid as part of comprehensive complication prevention.

Our study has several important strengths. We enrolled a consecutively recruited, clinic‐based sample with robust clinical phenotyping using the validated TCSS performed by trained clinicians with interobserver reliability checks, and we applied sex‐specific SUA thresholds to account for biologic differences. We adjusted for a comprehensive set of confounders, including renal function, and we evaluated discrimination using ROC analyses with Youden index–derived cutoffs. Importantly, this is among the first studies to report the SUA–DPN association in a northern Tanzanian tertiary clinic population, thereby adding crucial data from SSA where evidence remains sparse.

Despite these strengths, our findings must be interpreted in light of important limitations. The cross‐sectional design precludes causal inference and leaves open the possibility of reverse causation (e.g., that DPN or its comorbidities alter metabolism and SUA). Although we adjusted for measured confounders including eGFR, residual confounding by unmeasured factors (dietary purine intake, diuretic exposure, low‐grade inflammation markers, or cumulative glycemic exposure) may persist. Additionally, information on other diabetic microvascular and macrovascular complications was unavailable, potentially resulting in residual confounding. We relied on TCSS rather than electrophysiology, which is appropriate and pragmatic in low‐resource settings but limits direct electrophysiological characterization of neuropathy subtype. Our sample derived from a tertiary diabetes clinic and therefore likely overrepresents patients with longer disease duration and greater comorbidity relative to community samples, which may limit generalizability to primary care populations. Finally, while we used validated sex‐specific SUA cutoffs drawn from published literature, international thresholds vary, and optimal cutoffs for neuropathy risk require prospective validation.

## 5. Conclusion

In conclusion, in this Tanzanian study, sex‐specific hyperuricemia associated strongly and independently with prevalent DPN, alongside expected effects of duration, fasting hyperglycemia, adiposity, and low HDL. Our results add important African data to a heterogeneous global literature and provide biologic plausibility for further prospective and interventional research to determine whether SUA is a modifiable determinant of DPN. Future studies should prioritize longitudinal designs, careful medication and dietary measurement, biomarker panels of inflammation and oxidative stress, and, where feasible, randomized trials of urate‐lowering strategies in enriched high‐risk populations.

## Author Contributions

R.S.H.: conceptualization, data curation, formal analysis, investigation, methodology, and writing—original draft. W.P.H.: conceptualization, methodology, project administration, supervision, validation, visualization, and writing—review and editing. A.M.S.: conceptualization, formal analysis, methodology, project administration, supervision, validation, visualization, and writing—review and editing. I.A.L.: methodology, supervision, visualization, and writing—review and editing. H.F.A.: methodology, supervision, visualization, and writing—review and editing. E.W.M.: methodology, supervision, visualization, and writing—review and editing. K.G.K.: methodology, supervision, visualization, and writing—review and editing. F.S.L.: methodology, supervision, visualization, and writing—review and editing. G.B.N.: methodology, supervision, visualization, and writing—review and editing. E.R.S.: methodology, supervision, visualization, and writing—review and editing. D.P.M.: data curation, investigation, and writing—review and editing. D.F.B.: data curation, investigation, and writing—review and editing. N.M.: data curation, investigation, and writing—review and editing. P.O.: data curation, investigation, and writing—review and editing. N.N.: data curation, investigation, and writing—review and editing. L.L.: data curation, investigation, and writing—review and editing. H.L.M.: formal analysis, visualization, and writing—review and editing. N.G.C.: conceptualization, methodology, project administration, supervision, validation, visualization, and writing—review and editing.

## Funding

No funding was received for this manuscript.

## Disclosure

All authors agree to the final version and to be accountable for the content and conclusions within this article.

## Ethics Statement

The study was conducted in accordance with the Declaration of Helsinki. Ethical approval was obtained from the Kilimanjaro Christian Medical University College Research and Ethics Committee (Ethical Clearance Number PG 059/2024). All participants provided written informed consent prior to data collection.

## Conflicts of Interest

The authors declare no conflicts of interest.

## Data Availability

The data that support the findings of this study are available from the corresponding author upon reasonable request.
